# Biomimetic Synthesis of Nanocrystalline Hydroxyapatite Composites: Therapeutic Potential and Effects on Bone Regeneration

**DOI:** 10.3390/ijms20236002

**Published:** 2019-11-28

**Authors:** Chih-Hsiang Fang, Yi-Wen Lin, Feng-Huei Lin, Jui-Sheng Sun, Yuan-Hung Chao, Hung-Ying Lin, Zwei-Chieng Chang

**Affiliations:** 1 Institute of Biomedical Engineering, College of Medicine and College of Engineering, National Taiwan University, No. 1, Sec. 4, Roosevelt Rd, Taipei 10617, Taiwan; danny07291991@hotmail.com (C.-H.F.); zhew520@gmail.com (Y.-W.L.); double@ntu.edu.tw (F.-H.L.); 2 Division of Biomedical Engineering and Nanomedicine Research, National Health Research Institutes, No. 35, Keyan Road, Zhunan, Miaoli County 35053, Taiwan; 3 Department of Orthopedic Surgery, National Taiwan University Hospital, No. 7, Chung-Shan South Road, Taipei 10002, Taiwan; 4 Department of Orthopedic Surgery, College of Medicine, National Taiwan University, No. 1, Sec. 1, Ren-Ai Rd, Taipei 10051, Taiwan; 5 School and Graduate Institute of Physical Therapy, College of Medicine, National Taiwan University, No. 1, Sec. 1, Ren-Ai Rd, Taipei 10051, Taiwan; yuanhungchao@ntu.edu.tw; 6 School of Dentistry, College of Medicine, National Taiwan University, No. 1 Chang-Te Street, Taipei 10048, Taiwan; ephedrine0626@hotmail.com

**Keywords:** biomimetic, nano-hydroxyapatite, microsphere, stromal cell-derived factor-1, gelatin, alveolar bone defect

## Abstract

The development of a novel alloplastic graft with both osteoinductive and osteoconductive properties is still necessary. In this study, we tried to synthesize a biomimetic hydroxyapatite microspheres (gelatin/nano-hydroxyapatite microsphere embedded with stromal cell-derived factor-1: GHM-S) from nanocrystalline hydroxyapatites and to investigate their therapeutic potential and effects on bone regeneration. In this study, hydroxyapatite was synthesized by co-precipitation of calcium hydroxide and orthophosphoric acid to gelatin solution. The microbial transglutaminase was used as the agent to crosslink the microspheres. The morphology, characterization, and thermal gravimetric analysis of microspheres were performed. SDF-1 release profile and in vitro biocompatibility and relative osteogenic gene expression were analyzed, followed by in vivo micro-computed tomography study and histological analysis. The synthesized hydroxyapatite was found to be similar to hydroxyapatite of natural bone tissue. The stromal cell-derived factor-1 was embedded into gelatin/hydroxyapatite microsphere to form the biomimetic hydroxyapatite microsphere. The stromal cell-derived factor-1 protein could be released in a controlled manner from the biomimetic hydroxyapatite microsphere and form a concentration gradient in the culture environment to attract the migration of stem cells. Gene expression and protein expression indicated that stem cells could differentiate or develop into pre-osteoblasts. The effect of bone formation by the biomimetic hydroxyapatite microsphere was assessed by an in vivo rats’ alveolar bone defects model and confirmed by micro-CT imaging and histological examination. Our findings demonstrated that the biomimetic hydroxyapatite microsphere can enhance the alveolar bone regeneration. This design has potential be applied to other bone defects.

## 1. Introduction

In modern dentistry, implant therapy to restore an edentulous site has gained increasing popularity; while an adequate alveolar ridge dimensions to accommodate the implant is the prerequisite of a successful implant placement. Recent advances in tissue engineering have provided means for the effective regeneration of osseous defects. Bone regeneration can be accomplished by using different modalities; such as the autogenous bone graft, allografts, xenografts, and alloplasts [1]. Due to its excellent bioactivity, biocompatibility, high porosity, and osteo-conductivity, hydroxyapatites [HAP: Ca_10_(PO_4_)_6_(OH)_2_] is an ideal phase of calcium phosphate for application inside the human body. It has been successfully used for applications in the field of biomedical science ranging from bone regeneration to drug delivery. HAP is composed of ions commonly found in physiological environments, which makes it highly biocompatible [2]. Hydroxyapatites comprise about 50% of the weight of the bone, have excellent osteoconductive and osteointegrative properties. However, they do not possess any osteo-inductive properties [3].

Gelatin is commonly used for pharmaceutical and medical applications [4]. Indeed, the gelatin hydrogel or sponge can control the release of growth factors and then enhance their biological functions in bone regeneration [5,6]. In vivo, the complexed gelatin/biomolecule fragments are released by enzymatic degradation; the density of cross-linking within gelatin hydrogels can affect their degradation rate and the rate of biomolecule release from gelatin carriers [7,8].

In the ideal environment for bone healing, the osteo-conductive bone substitutes will facilitate the migration and attachment of progenitor cells. When this situation is disturbed, the situation will predispose to be delayed union or even non-union. At such situation, the presence of osteo-inductive factors at the bone healing site becomes critically important [9,10]. In treating skeletal defects, bone morphogenetic proteins (BMPs), especially BMP-2 and BMP-7, are the most potent osteo-inductive factors studied and have accrued the most considerable evidence of efficacy [11]. However, the application of these growth factors is limited by several drawbacks. BMPs have a short biological half-life, rapid metabolism, and potentially harmful side-effects, such as cancer formation [12].

Stromal cell-derived factor is a chemokine that is involved in wound healing [13], tissue repair [14] and immune cell activation, differentiation, and migration [15]. Stromal cell-derived factor-1 (SDF-1) is also a strong candidate for promoting regeneration as it is a well-characterized chemokine for attracting stem cells. Previous studies also report that stromal cell-derived factor-1 (SDF-1) promotes stem cells survival and development [16], and regulated osteogenic differentiation [17]. Recent study has demonstrated that angiogenesisis also closely associated with SDF-1 and this is essential for osteogenesis [18].

Specific surface area and adsorption capacity strongly increase as the particle decreases to the scale of nano-size. Chemical and physical processes that take place on their surface strongly affect the properties of particles [19]. Although hydroxyapatite nanoparticles show a great promise for medical applications; there are still concerns about the safety of using these materials in biological environments. Recently, the nephron-toxicity for HAP nanoparticles had been raised [20]. Previous studies have also shown that metallic implants coated with nano-sized hydroxyapatite had better osseointegration than those metallic implants coated with conventional or micron-sized hydroxyapatite particles. As cell proliferation is a prerequisite for bone formation, amphiphilic peptides that promote osteoblasts attachment and enhance other cell functions could improve osteoblast functions. The addition of amphiphilic peptide to nano-hydroxyapatites can induce greater osteoblast densities and then bone formation [21]. The combination of biodegradable polymers and bioactive inorganic materials is a promising method to mimic native tissue in bone tissue regeneration. Considering the ever-growing number of hydroxyapatite nanoparticle applications, we decided to investigate the possible applications of hydroxyapatite nanoparticles. In this study, we tried to synthesize biomimetic hydroxyapatite microspheres (gelatin/nano-hydroxyapatite microsphere embedded with stromal cell-derived factor-1: GHM-S) from nanocrystalline hydroxyapatites and to investigate their therapeutic potential and effects on bone regeneration.

## 2. Results

### 2.1. Material Characteristics and Biocompatibility of the Biomimetic Hydroxyapatite Microspheres (GHM-S)

**Figure 1:**
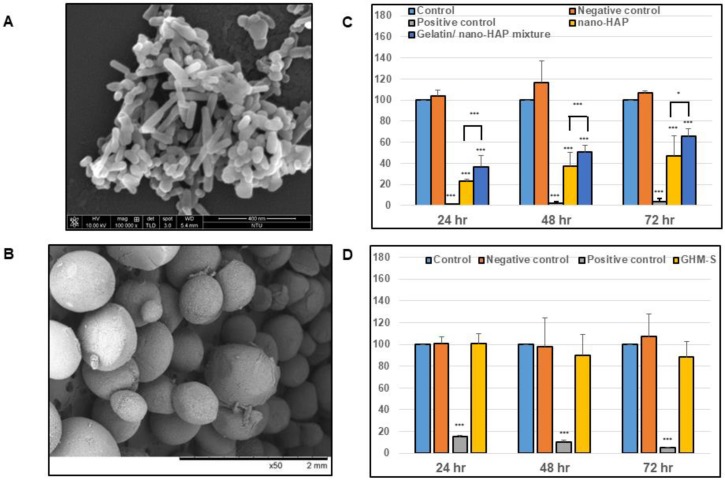
Scanning electron microscopy morphology and evaluation of mouse 3T3 viability by WST-1 assay. The in vitro biocompatibility was evaluated according to the ISO 10993-5 standard. (**A**) Scanning electron microscopy (SEM) images nanocrystalline hydroxyapatite particles (nano-HAP). Bar = 400 nm. (**B**) Scanning electron microscopy (SEM) images biomimetic nanocrystalline hydroxyapatite composites (GHM-S: gelatin/nano-hydroxyapatite microsphere embedded with stromal cell-derived factor-1). Bar = 2 mm. (**C**) Biocompatibility test for nanocrystalline hydroxyapatite particles (nano-HAP). (**D**) Biocompatibility test for biomimetic nanocrystalline hydroxyapatite composites. Note: The biomimetic hydroxyapatite microspheres (GHM-S) was synthesized by gelatin/nano-hydroxyapatite microsphere embedded with stromal cell-derived factor-1. (*n* = 12; * *p* < 0.05; *** *p* < 0.001).

Scanning electron microscopy (SEM) examination are performed on both nanocrystalline hydroxyapatite particles (nano-HAP) and biomimetic nanocrystalline hydroxyapatite composites (GHM-S: gelatin/nano-hydroxyapatite microsphere embedded with stromal cell-derived factor-1). The size of nanocrystalline hydroxyapatite particles (nano-HAP) is about 50–100 nm (Figure 1A) and the size of biomimetic nanocrystalline hydroxyapatite composites (GHM-S) is at 300–500 μm (Figure 1B). The in vitro biocompatibility was evaluated according to the ISO 10993-5 standard. The cell viability of the hydroxyapatite nanocrystals and biomimetic hydroxyapatite microspheres (GHM-S: experimental groups) was tested on 3T3 cells in a WST-1 assay. The cell viability was measured on days 1 to 3, with the results expressed as a percentage of cell viability. The cell viability of nano-hydroxyapatite particles is significantly decreased; the potential cytotoxic effect of nano-hydroxyapatite particles can be alleviated by gelatin (Figure 1C). For the biomimetic hydroxyapatite microspheres used in this study, the cell viability of the experimental groups (GHM-S) did not show any significant change when compared to the control groups (Figure 1D). These results confirm that the biomimetic hydroxyapatite microspheres (GHM-S) did not have cytotoxic effect or inhibit cell proliferation in 3T3 fibroblasts.

### 2.2. Material Characteristics of Gelatin/Hydroxyapatite Microsphere (GHM)

**Figure 2:**
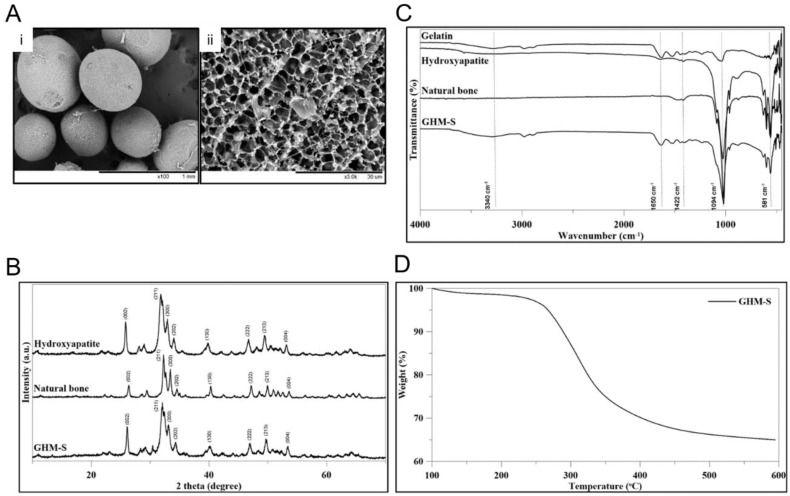
Material characteristics of biomimetic hydroxyapatite microspheres (gelatin/hydroxyapatite microsphere embedded with stromal cell-derived factor-1: GHM-S). (**A**) Scanning electron microscopy (SEM) images of biomimetic hydroxyapatite microspheres (GHM-S) at different magnifications: (**i**) 100× and (**ii**) 3000×. The images show an open and interconnected porous structure with homogeneous pores in the biomimetic hydroxyapatite microspheres (GHM-S). Bar = 1 mm (100×) and 30 μm (3000×). (**B**) The X-ray diffractometer (XRD) patterns of conventional hydroxyapatite, natural bone tissue, and biomimetic hydroxyapatite microspheres (GHM-S). The GHM demonstrated broad diffractions corresponding to (002), (211), (300), (202), (130), (002), (222), and (213) of the conventional hydroxyapatite. The results confirmed the formation of HAP mineralization. (**C**) The Fourier-transform infrared spectroscopy (FTIR) spectrum of conventional hydroxyapatite, natural bone tissue, and biomimetic hydroxyapatite microspheres (GHM-S). The characteristic peaks for hydroxyapatite were located in the 600–1100 cm^−1^ region. The asymmetric bending and the stretching band of the (PO_4_)^3−^ group was found at 1063 cm^−1^. In addition, characteristic peaks for gelatin were observed at 2800–2950 cm^−1^ (C-H stretching), 1652 cm^−1^ (C=O group), and 3420 cm^−1^ (N-H stretching), respectively. (**D**) Thermogravimetric analysis (TGA) of the biomimetic hydroxyapatite microspheres (GHM-S). As monitored by TGA analysis, a significant weight loss occurred between 300 and 400 °C due to the burn-out of the polymeric phase (gelatin and SDF-1 protein) of the biomimetic hydroxyapatite microspheres (GHM-S) is shown. Note: gelatin/nano-hydroxyapatite microsphere embedded with stromal cell-derived factor-1 (GHM-S). Material characteristics of gelatin/hydroxyapatite microsphere (GHM); Scanning electron microscopy (SEM) images of gelatin/hydroxyapatite microsphere (GHM). The gelatin/hydroxyapatite microsphere (GHM) had a particles size between 150 μm and 2000 μm; with a mean size of 358.9 ± 197.6 μm.

As shown in the scanning electron microscopy (SEM), the gelatin/nano-hydroxyapatite microsphere (GHM) had a particles size between 300 μm and 500 μm with a particle surface pore size of 3 μm. The images show an open and interconnected porous structure with homogeneous pores in the gelatin/hydroxyapatite microsphere (GHM) (Figure 2A). The XRD pattern of the biomimetic hydroxyapatite microspheres (GHM-S) was similar to conventional hydroxyapatite of natural bone. The GHM-S demonstrated broad diffractions corresponding to (002), (211), (300), (202), (130), (002), (222), and (213) of the conventional hydroxyapatite. The results confirmed the formation of HAP mineralization (Figure 2B). The characteristic peaks for hydroxyapatite were located in the 600–1100 cm^−1^ region. The asymmetric bending and the stretching band of the (PO_4_)^3−^ group was found at 1063 cm^−1^. In addition, characteristic peaks for gelatin were observed at 2800-2950 cm^−1^ (C-H stretching), 1652 cm^−1^ (C=O group), and 3420 cm^−1^ (N-H stretching), respectively. The infrared spectra of the biomimetic hydroxyapatite microspheres (GHM-S) showed characteristic peaks corresponding to gelatin and hydroxyapatite (Figure 2C). As monitored by TGA analysis, a significant weight loss occurred between 300 and 400 °C due to the burn-out of the polymeric phase (gelatin and SDF-1 protein) of the biomimetic hydroxyapatite microspheres (GHM-S) was shown in Figure 2D.

### 2.3. SDF-1 Releasing Profile

**Figure 3:**
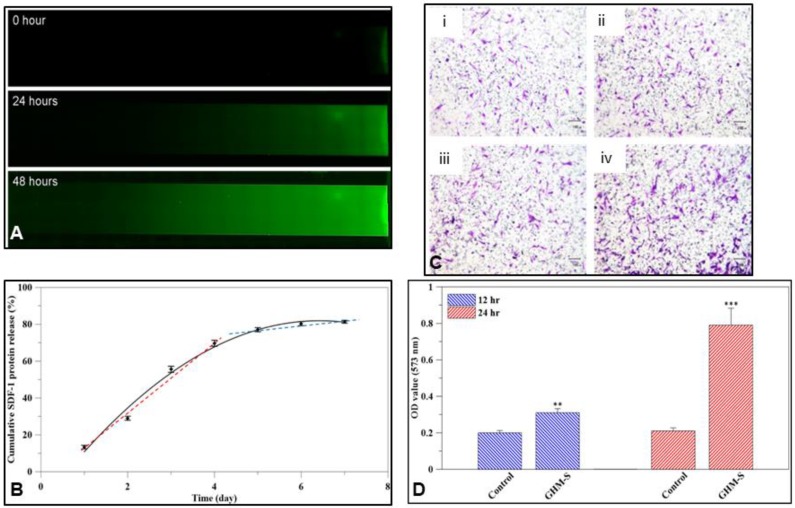
SDF-1 releasing profile and cell attracting test for the biomimetic hydroxyapatite microspheres (GHM-S). (**A**) Diffusion of FITC labeled SDF-1 protein in the μ-slide at different time periods. After 24 h of release, the FITC labeled SDF-1 had diffused to half of the μ-slide, then over the whole μ-slide after 48 h. (**B**) The cumulative amount of SDF-1 protein released from the GHM-S. The cumulative amount of SDF-1 protein release indicated that an increasing amount of SDF-1 protein was released from the biomimetic hydroxyapatite microspheres (GHM-S) over time. (**C**) Transwell migration assay. With SDF-1 protein, the hMSC significantly migrated to the lower chamber after 12 and 24 h co-culture. (**i**) Macroscopic observation of transwell chamber incubation 12 h (without GHM-S). (**ii**) Macroscopic observation of transwell chamber incubation 12 h (with GHM-S). (**iii**) Macroscopic observation of transwell chamber incubation 24 h (without GHM-S). (**iv**) Macroscopic observation of transwell chamber incubation 24 h (with GHM-S). (**D**) The OD value of crystal violet after 12 and 24 h migration. The absorption at 573 nm following treatment with SDF-1 was significantly increased in comparison to the control group (without GHM-S). Note: SDF-1 releasing profile from the biomimetic hydroxyapatite microspheres (GHM-S). Gelatin/hydroxyapatite microsphere embedded with stromal cell-derived factor-1 (GHM-S). (*n* = 12; ** *p* < 0.01; *** *p* < 0.001).

FITC labeled SDF-1 protein was released from the biomimetic hydroxyapatite microspheres (GHM-S) and formed a concentration gradient of SDF-1 protein. After 24 h of release, the FITC labeled SDF-1 had diffused to half of the μ-slide, then over the whole μ-slide after 48 h (Figure 3A); the SDF-1 protein was released from the biomimetic hydroxyapatite microspheres (GHM-S) over time (Figure 3B). With SDF-1 protein, the hMSC significantly migrated to the lower chamber after 12 and 24 h co-culture (Figure 3C). Moreover, the absorption at 573 nm following treatment with SDF-1 was significantly increased in comparison to the control group (Figure 3D).

### 2.4. Gene Expression and Quantitative Analysis of Osteogenic Proteins

**Figure 4:**
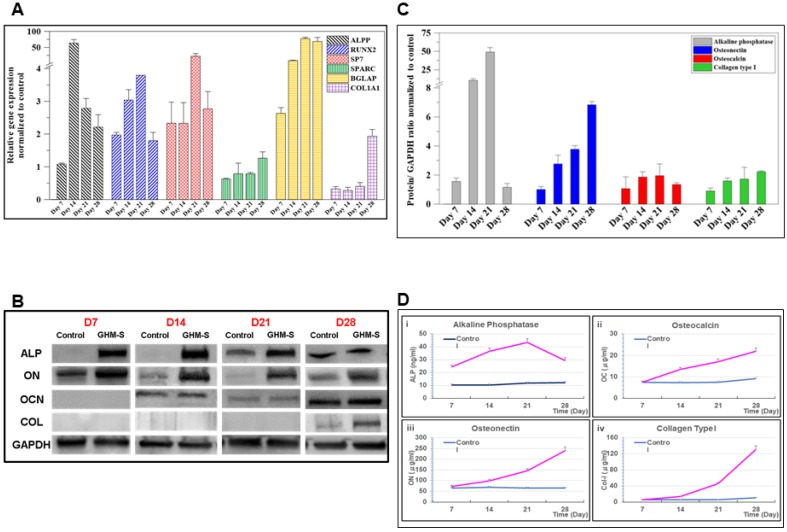
Gene expression and quantitative analysis of osteogenic proteins. (**A**) The results of relative osteogenic gene expression from hMSCs cultured with the biomimetic hydroxyapatite microspheres (GHM-S) and normalized to the control group. The osteoblast-specific genes of the hMSCs, including alkaline phosphatase (ALPP), osteocalcin (BGLAP), type I collagen (COL1a1), osteonectin (SPARC), osterix (SP7), and runt-related transcription factor-2 (RUNX-2) were upregulated compared to the control group. (**B**) Western blotting of osteogenic protein after hMSCs co-culture with the biomimetic hydroxyapatite microspheres (GHM-S) for four weeks. The hMSCs co-cultured with the biomimetic hydroxyapatite microspheres (GHM-S) for four weeks showed osteogenic protein overexpression at different time periods compared to the control group. (**C**) The amount of western blotting product in hMSCs was taken as 1.0, and the relative ratio of the products is indicated in the ordinate. The value was corrected using the expression of GAPDH as an internal control. Alkaline phosphatase was overexpressed at the early stage, followed by overexpression of osteocalcin and osteonectin at the medium to later stages, and, finally, collagen type I expression in the later stage. (**D**) ELISA kits were used to analyze the osteo-specific protein expression of hMSCs cultured with the biomimetic hydroxyapatite microspheres (GHM-S). The osteoblast specific-proteins of hMSCs, including alkaline phosphatase, osteonectin, osteocalcin, and collagen type I, were upregulated compared to the control group. (**i**) concentration of alkaline phosphatase at 7, 14, 21, and 28 days; (**ii**) concentration of osteocalcin at 7, 14, 21, and 28 days; (**iii**) concentration of osteonectin at 7, 14, 21, and 28 days; (**iv**) concentration of collagen type I at 7, 14, 21, and 28 days. The osteoblast-specific proteins of hMSCs, including ON, OC, and collagen type I, were upregulated in comparison to the control group (without biomimetic hydroxyapatite microspheres). Otherwise, the OC and ON overexpression at the medium to later stages, and COL expression at the later stage. Note: gelatin/hydroxyapatite microsphere embedded with stromal cell-derived factor-1 (GHM-S); (*n* = 6, ** *p*< 0.01; *** *p* < 0.001).

The osteo-differentiation of the hMSCs was accompanied by a cascade of intracellular changes in gene and protein expression. As shown in Figure 4A, the osteo-differentiation of the hMSCs, several genes (type I collagen (COL1a1), runt-related transcription factor-2 (RUNX-2), alkaline phosphatase (ALPP), osterix (SP7), osteonectin (SPARC), and osteocalcin (BGLAP)) were upregulated when compared to the control samples; while osteogenic proteins expression were also enhanced at different time periods (Figure 4B). The relative intensity was normalized to an internal control, GAPDH, and the control group using ImageJ software. Alkaline phosphatase was over-expressed at the early stage, followed by over-expression of osteocalcin and osteonectin at the medium to later stages, and, finally, collagen type I expression in the later stage (Figure 4C). ELISA kits were used to analyze osteo-specific protein expression from MSCs cultured with the MGS microsphere over four weeks; as shown in Figure 4D, the osteoblast-specific proteins of hMSCs, including ON, OC, and collagen type I, were up-regulated in comparison to the control group (Figure 4D).

### 2.5. Micro-Computed Tomography (μ-CT) Analysis

**Figure 5:**
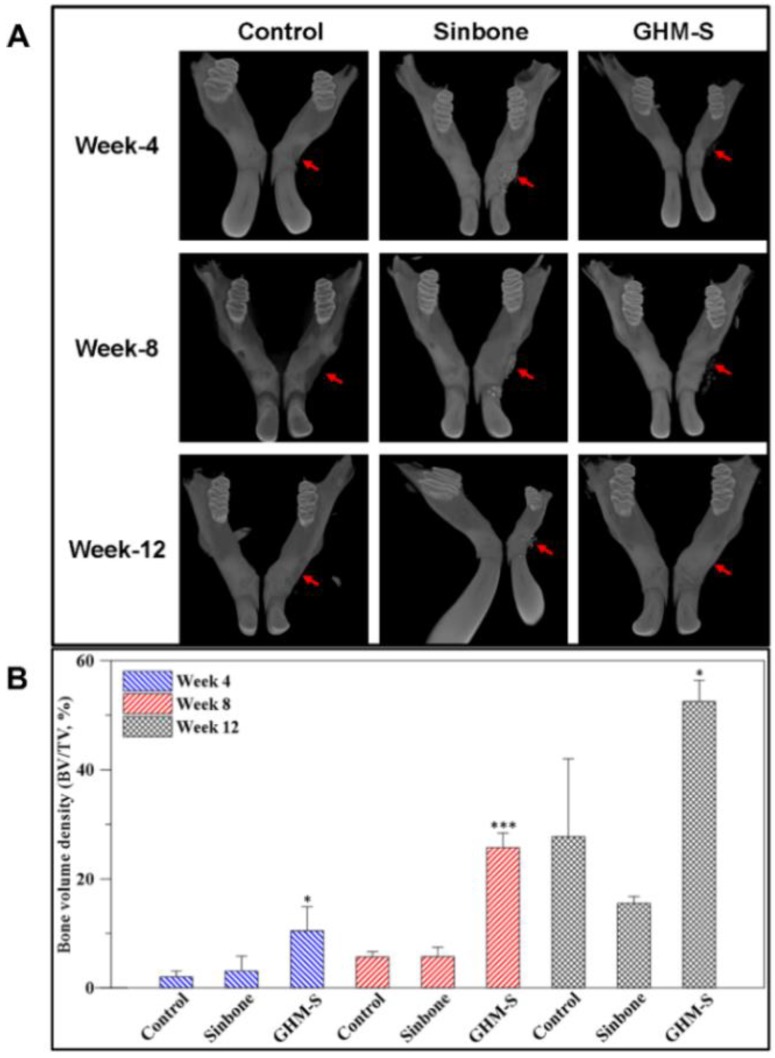
Micro-computed tomography (μ-CT) analysis. (**A**) Three dimensional (3D) μ-CT image analyses of the control and experimental groups. 3D μ-CT tomographs of the control, Sinbone, and biomimetic hydroxyapatite microspheres (GHM-S) groups present the defect area and new bone growth 4, 8, and 12 weeks post-implantation. Red arrow indicates a defect area and bone in-growth after filling with bone substitutes. (**B**) Analysis of bone volume density by µ-CT tomography at different time points. The results of control group (defect site with no implant) showed limited regeneration. The bone formation in the Sinbone and the biomimetic hydroxyapatite microspheres (GHM-S) samples showed increased bone growth over time. The defect area filled with the biomimetic hydroxyapatite microspheres (GHM-S) showed significant alveolar bone regeneration after 12 weeks. The Sinbone granules showed a lack of complete mineralization even after 12 weeks, however still occupied the defect site, thus affecting the new bone formation. Note: gelatin/nano-hydroxyapatite microsphere embedded with stromal cell-derived factor-1 (GHM-S); (*n* = 3, ** *p* < 0.01; *** *p* < 0.001).

The in vivo high-resolution 3D micro-computed tomography (μ-CT) was used to quantitatively examine alveolar bone healing in the experimental and control groups at 4, 8, and 12 weeks after implantation. These samples were then imaged and analyzed using 3D μ-CT (Figure 5A). The difference in hard tissue volume (%) was calculated from μ-CT using quantitative analysis software. The control group (defect site with no implant) showed limited regeneration. The bone formation in the Sinbone and biomimetic hydroxyapatite microspheres (GHM-S) samples showed increased bone growth over time. The defect area filled with the biomimetic hydroxyapatite microspheres (GHM-S) showed significant alveolar bone regeneration after 12 weeks; while the Sinbone granules showed a lack of complete mineralization even after 12 weeks (Figure 5B).

### 2.6. Histological and Immunohistochemical Analysis

**Figure 6:**
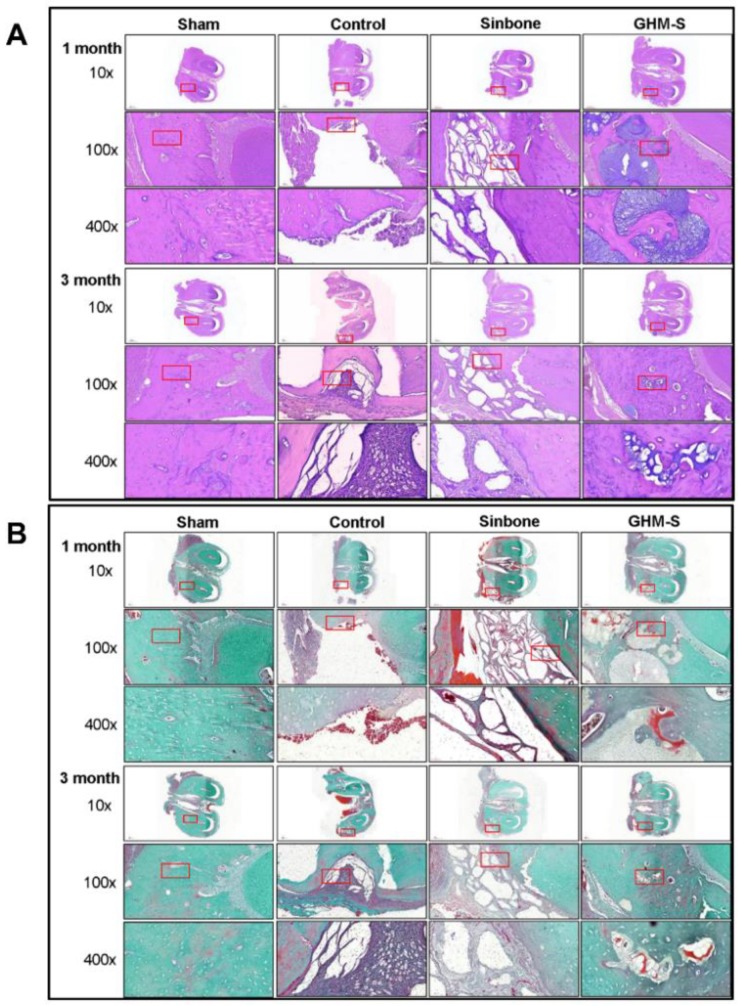
Histological and Immunohistochemical analysis. (**A**) Hematoxylin and eosin (H&E) staining. (**B**) Goldner’s Trichrome staining. Trabecular bone formation in the control group was not significantly developed and the defect site was mostly filled with fibrous connective tissue. The Sinbone granules did not degrade and remained at the defect site 12 weeks after implantation. In contrast, active new bone formation was observed in the defect site treated with biomimetic hydroxyapatite microspheres (GHM-S) implants, with the presence of Harversian canals within the newly formed bone. In addition, there was no detectable fibrous tissue invasion or inflammatory cell infiltrates in the biomimetic hydroxyapatite microspheres (GHM-S) experimental group. Note: gelatin/nano-hydroxyapatite microsphere embedded with stromal cell-derived factor-1 (GHM-S).

New bone formation in the control, Sinbone, and MGS samples was evaluated after 4 weeks and 12 weeks post-implantation by histological analysis. After 4 weeks and 12 weeks post-implantation, all the experimental groups showed new bone formation or tissue-in growth within the defect site, confirmed by H&E staining (Figure 6A) and Goldner’s Trichrome staining (Figure 6B). Trabecular bone formation in the control group was not significantly developed and the defect site was mostly filled with fibrous connective tissue. The Sinbone granules did not degrade and remained at the defect site 12 weeks after implantation. In contrast, active new bone formation was observed in the defect site treated with biomimetic hydroxyapatite microspheres (GHM-S) implants, with the presence of Harversian canals within the newly formed bone. In addition, there was no detectable fibrous tissue invasion or inflammatory cell infiltrates in the biomimetic hydroxyapatite microspheres (GHM-S) experimental group.

## 3. Discussion

Nowadays, although there are many techniques that allows restoring an appropriate alveolar bone thickness, but their attention is mainly focused on the use of natural or synthetic grafts [22]. In this study, we try to develop a biomimetic hydroxyapatite microspheres (gelatin/nano-hydroxyapatite microsphere embedded with stromal cell-derived factor-1: GHM-S) to attract stem cells and also to promote their osteogenic differentiation for alveolar bone regeneration.

Although hydroxyapatite nanoparticles show a great promise for medical applications due to their unique properties at the nanoscale. Controversy about the impact of the physico–chemical properties of HAP nanoparticles and their aggregates (or agglomerates) on the living cells is not yet fully understood. At the nanoscale level, HAP nanoparticles present marked emergent properties differing substantially from those of the bulk counterpart; which may be cytotoxic and anti-proliferative on the living cells [23]. Our study also showed that nano-sized HAP particles may exert cytotoxic and anti-proliferative effect on the hMSC cells (Figure 1C). Significant differences in these properties as a result from different manufacturing methods should be taken into consideration; lower crystallinity, high purity of hydroxyapatite nanoparticles are more biocompatible for long-term use in the body [24].

In previous work focused on the development of gelatin-based scaffolds crosslinked by carbodiimide and their bioactivation by hydroxyapatite nanoparticles or BMP-2 analog; Raucci et al. demonstrated that the presence of bioactive signals (either inorganic or organic) at nanoscale level allowed an osteo-inductive effect on hMSCs [25]. In this study, the newly synthesized gelatin/nano-hydroxyapatite microspheres (GHM) demonstrated the incorporation of hydroxyapatite into the gelatin fibril which was similar to that of the natural bone (Figure 2B,C) [26]. High HA solid content (40 wt%) in the HA pure scaffolds was reported to be negative for cell viability and proliferation [27]; the ratio between organic and inorganic components (wt% = 37:63) of biomimetic hydroxyapatite microspheres (GHM-S) was similar to that found in natural bone tissue (Figure 2D) [28] and did not show any cytotoxic effect on the hMSCs (Figure 1D).

Previous studies showed that SDF-1α physically loaded onto gelatin electrospun membranes yielded a 6-fold higher amount of bone formation [29]. In this study, the SDF-1 protein releasing profile was a high initial burst, followed by a later slow release (Figure 3A,B). As was reported in previous studies, microspheres absorb high amounts of water from the medium and become noticeably swollen. After reaching the swelling equilibrium, disintegration begins, followed by degradation. As the mTG-crosslinking was performed in this study, the degradation and drug release rate of the microspheres can be slowed down [30]. The bonding strategy for SDF-1 incorporation into the GHM-S scaffolds can be classified as physical and chemical immobilization. This type of release behavior is preferred for the biomimetic hydroxyapatite microspheres (GHM-S) as an initial burst can produce an immediate therapeutic effect, followed by a prolonged drug release to maintain the treatment effect (Figure 3B) [31].

SDF-1 is a key stem cell-homing factor that mobilize of stem cells from the bone marrow to the peripheral blood and for subsequent engraftment in the tissues of diseased organs. The endogenous SDF-1 only recruits a small number of stem cells adjacent to the defect area, whereas a high concentration of exogenous SDF-1 (in addition to endogenous SDF-1) is likely to recruit more circulating and local stem cells to participate in healing [32]. Furthermore, the gradients of exogenous SDF-1 concentration can lead the mobilization and homing of stem cells [33]. The production of endogenous SDF-1 can be induced by injury itself, but its production is usually limited with relatively low concentration. Although the MSCs and HSCs were engrafted widespread throughout the whole bone defect after SDF-1 treatment, they still scattered adjacent to the border of the defect and only few stem cells were observed in the central regions of the specimens [34]. Our findings show that SDF-1 enhances MSC migration and that the extent and length of MSC recruitment depends heavily on the duration of SDF-1 release (Figure 3C,D). Osteogenic proteins, including alkaline phosphatase, osteonectin, osteocalcin, and collagen type I, are essential for osteogenesis and their upregulation is a positive indicator of osteogenesis. Here, the hMSCs co-cultured with the GHM-S microsphere for four weeks showed osteogenic protein overexpression at different time periods compared to the control group (Figure 4).

The CXCR4 is a unique chemokine receptor for SDF-1; while periodontal ligament stem cells (PDLSCs) can express CXCR4 and that SDF-1 also promotes their viability, chemotaxis, proliferation, and differentiation [35]. The SDF-1/CXCR4 may play a dual role in inflammation and tissue repair. In vitro, SDF-1 showed chemotactic properties for stem cells; but the in vivo environment is more complex. Liu et al. reported SDF-1 may have a potential role in stem cell recruitment, inflammatory responses, and bone repair [36]. In the absence of injury, exogenous SDF-1 is not sufficient to induce stem cell recruitment [37]; while in the presence of inflammatory stimuli, SDF-1 can recruit more stem cells to participate in healing [38]. These contradictory results indicate that complex interactions of SDF-1 may exist in stem cell recruitment and inflammatory responses. The success of the dental implants is often limited by the occurrence of inflammation and its consequences during the post-operation period. Hence, the prevention of a chronic inflammatory response or osteogenic cell stress in response to the implant-derived wear particles can enhance the overall success rate of the implants [39]. In this study, the drug release profile of the microsphere showed a significant change in SDF-1 drug bioavailability and possibly its therapeutic efficacy; the high initial SDF-1 burst is likely to be involved in the recruitment of stem cells in vivo, whereas the later slow release is related to decreases the density of inflammatory cells and hence to prevent a chronic inflammatory response and to enhance the overall success rate of the implants.

The chemotactic properties of SDF-1 are mediated by interactions with its receptor, CXCR4, while both mesenchymal stem cells (MSCs) and hematopoietic stem cells (HSCs) express CXCR4 [40,41]. In this study, we demonstrated the positive clinical outcomes of the biomimetic hydroxyapatite microspheres (GHM-S) as the bone regeneration was augmented. The quantitative 3D μ-CT analysis and histological staining suggested that the formation of new bone in the defect area was greatly enhanced (Figure 5); Goldner’s trichrome staining also showed that a significantly new bone and angiogenesis were also found in the biomimetic hydroxyapatite microspheres (GHM-S) sample (Figure 6). By using an in vivo rat mandibular bone defect model, Liu et al. reported that SDF-1 not only accelerated degradation of collagen membrane scaffolds, thus providing space for newly formed tissues, but also decreased infiltration by inflammatory cells and alleviated inflammation; they suggested that exogenous SDF-1 facilitated vascular formation and promoted bone regeneration [34]. The regenerating callus is highly metabolically active and angiogenesis is a key component of bone repair. New blood vessels can bring nutrients and oxygen and also serve as the route for cells to reach the injury site [42]. By improving their microenvironment, the endothelial progenitor cells (EPCs) may also enhance the osteogenic properties of MSCs; transplantation of mesenchymal stem cells (MSCs) may promote bone healing. This is in consistent of our results on quantitative 3D μ-CT analysis, histological staining and Goldner’s trichrome staining.

## 4. Materials and Methods

### 4.1. Materials

Calcium hydroxide (Ca(OH)_2_, Cat. No. 1305-62-0), hydrochloric acid (HCl, No. 7647-01-0), and gelatin (Cat. No. 9000-70-8) were obtained from Sigma-Aldrich (St. Louis, MO, USA). Orthophosphoric acid (H_3_PO_4_, Cat. No. 7664-38-2) was obtained from Baker (Center Valley, PA, USA). Sodium hydroxide (NaOH, Cat. No. 1310-73-2) was obtained from SHOWA (Meguroku, Tokyo, Japan). Recombinant human SDF-1α was obtained from Peprotech (Rocky Hill, NJ, USA). Microbial transglutaminase (mTGase) was obtained from Activa (Ajinomoto, Japan).

### 4.2. Fabrication of Gelatin/Hydroxyapatite Microsphere (GHM) and Biomimetic Hydroxyapatite Microspheres (GHM-S)

For nano-hydroxyapatite mineralization process on gelatin fibrils, nano-hydroxyapatite was synthesized in situ by co-precipitation of calcium hydroxide and orthophosphoric acid. Briefly, 3.665 g of calcium hydroxide was added to 95 mL 3.7 wt% gelatin solution. A stoichiometric quantity of 0.15 M orthophosphoric acid solution (1.95 mL, at 85 °C) was added drop-wise at a rate of 0.3 mL/min and then magnetically stirred for 24 h. First, the pH was then adjusted to 9 by using 1 N NaOH solution to ensure that pure nano-HAP precipitation can be formed with the desired morphology; then to a neutral level. These amounts of starting materials were determined from the calculation that the expected weight ratio of gelatin/hydroxyapatite was 1:1. The microspheres were prepared by spattering into liquid hexane on an ice bath. Finally, 0.1% microbial transglutaminase (mTGase) was used as a cross-linking agent to crosslink the microspheres (GHM). The biomimetic hydroxyapatite microspheres (GHM-S) was synthesized by gelatin/nano-hydroxyapatite microsphere embedded with stromal cell-derived factor-1. For the synthesis of GHM-S, 200 μg SDF-1 was added to the mixture before spattering into liquid hexane on an ice cooling bath and cross-linked by 0.1% microbial transglutaminase (mTGase). After evaporation of hexane, the specimens were moved to 4 °C for 24 h, frozen at −20 °C for 24 h, −80 °C 24 h, and then lyophilized for 72 h.

### 4.3. Morphology of Gelatin/Nano-Hydroxyapatite Microsphere (GHM)

The gelatin/hydroxyapatite microsphere (GHM) specimens were sputtered with gold coating and analyzed by using a Hitachi S3000/N electron microscope (Hitachi, Ltd., Tokyo, Japan).

### 4.4. Characterization of Gelatin/Nano-hydroxyapatite Microsphere (GHM)

X-ray diffractometry is a rapid analytical technique primarily used for the phase identification of a crystalline material which provides information of the unit cell dimensions. An X-ray diffractometer (Rigaku Corporation, The Woodlands, TX, USA) was used to determine the crystal structure. The previously prepared powders were mounted on the sample holder of the X-ray powder diffractometer under Cu KαI (λ = 0.15406 nm) radiation, using a Ni filter with a potential of 30 kV at a current of 15 mA. The specimen was scanned at a range from 10° to 70° and a speed of 2°/min. The patterns were analyzed using a model auto-matched to the international center for the diffraction database by using Jade 6.0 software (Materials Data Inc., Livermore, CA, USA).

During infrared excitation, different molecules will produce different types of vibration at a specific frequency. The principle is that the dipole moment of the molecule is changed, such that Fourier-transform infrared spectroscopy can be used to analyze the functional groups of the organic compounds. The Fourier transform infrared (FTIR) spectra of the different synthesized materials, including pure gelatin, pure hydroxyapatite, and the MGS microsphere, were collected for analysis. For the FTIR measurements, a spectrophotometer was used. The spectra were recorded at wavelengths ranging from 450 to 4000 cm^−1^. The characterization of the MGS microsphere was then compared to that of the natural bone as a control.

### 4.5. Thermogravimetric Analysis (TGA) of Biomimetic Hydroxyapatite Microspheres (GHM-S)

Thermogravimetric analysis or thermal gravimetric analysis (TGA) is a method of thermal analysis in which the mass of a sample is measured over time as the temperature changes. In this study, we used thermal gravimetric analysis (TGA) to compare the percent weight loss of organic component, such as gelatin and SDF-1 protein, at a temperature 500 °C, to confirm the organic component in the biomimetic hydroxyapatite microspheres (GHM-S: gelatin/nano-hydroxyapatite microsphere embedded with stroma-cell derived factor-1(SDF-1)).

### 4.6. SDF-1 Release Profile and Gradient Formation

FITC-labeled SDF-1 (Infinite^®^ 200 PRO, Tecan, Redmond, WA, USA) was used for the fabrication of the biomimetic hydroxyapatite microspheres (GHM-S). The profile and gradient of SDF-1 protein release was measured using the FluoReporter^®^ FITC Protein Labeling Kit (Thermo Scientific, Waltham, MA, USA) and determined by fluorescence microscopy at the different time periods.

### 4.7. In Vitro Study

The experimental protocol received prior approval from the Institutional Review Board, National Taiwan University Hospital (NTUH IRB No. 201704005 RINA). Human mesenchymal stem cells (hMSCs) were collected from bone marrow aspirate during total hip/knee joint replacement surgery. Mononuclear cells (MNC) fraction were isolated according to standard techniques using Ficoll-Paque PLUS (GE Healthcare, Amersham, UK). In this study, the hMSCs from passages 3–4 were used for the subsequent experiments.

The biocompatibility of the biomimetic hydroxyapatite microspheres (GHM-S) was evaluated using a WST-1 assay with mesenchymal stem cells according to the ISO 10993-5 standard. The migration assay was designed using transwell plates (Corning Costar, Cambridge, MA, USA). In the chemotaxis assay, the upper chambers were loaded with 5 × 10^4^ hMSCs in 200 μL of DMEM containing 0.1% BSA, and the lower chambers with 500 μL of DMEM containing 10% FBS and the biomimetic hydroxyapatite microspheres (GHM-S) or the control sample. The cells that had migrated to the lower side of the filter were fixed, stained, and measured at 573 nm wavelength with an enzyme-labeling measuring instrument.

The relative expression fold changes of five bone-related genes, including runt-related transcription factor 2 (Runx2), collagen type 1 (COL1), alkaline phosphatase (ALP), osterix (SP7), osteonectin (ON or SPARC), and osteocalcin (OC or BGLAP), were quantified using real-time RT-PCR at days 7, 14, 21, and 28 after seeding cells with the biomimetic hydroxyapatite microspheres (GHM-S). The osteo-specific primers (Tools Co., Taichung, Taiwan) used for osteogenic differentiation are shown in Table 1. Qiazol (Qiagen, Valencia, CA, USA) was used for total RNA extraction, according to the manufacturer’s protocol. Random hexamers (Vivantis Inc., CA, USA) and reverse transcriptase (Vivantis Cat No: RTPL12; Vivantis Inc., CA, USA) were used for the first-strand cDNA synthesized with the following PCR parameters: 95 °C for denaturation (3 min), 40 cycles of 95 °C for 20 s, 60 °C for annealing (30 s), and 72 °C for elongation (30 s). SYBR Premix-Ex-Taq Master-mix (TaKaRa Biotechnology, Dalian, China) was applied for real-time RT-PCR using Rotor-Gene 6000 (Corbett, Concorde, NSW, Australia). The expression of the target genes was calculated in using glyceraldehyde 3-phosphate dehydrogenase (GAPDH) as an endogenous control.

Three samples from each group were washed, lysed, and equal amounts of protein were separated and transferred to a PVDF membrane (Millipore, Burlington, MA, USA), blocked and incubated with primary antibodies against osteocalcin (ab13418), osteonectin (ab14174), collagen type I (ab229389), alkaline phosphatase (ab83259), and glyceraldehyde 3-phosphate dehydrogenase (ab8245; Abcam, 1:1000). The proteins were visualized by enhanced chemiluminescence (ECL). The relative intensity was normalized to an internal control, GAPDH, and the control group using ImageJ software (developed at the National Institutes of Health and the Laboratory for Optical and Computational Instrumentation (LOCI, University of Wisconsin, Madison, WI, USA).

### 4.8. In Vivo Study

All animal experiments were carried out in compliance with the National Taiwan University, College of Medicine, Institutional Animal Care and Use Committee (IACUC). The experimental protocol received prior approval by the Institutional Animal Care and Use Committee (IACUC) (No. 20180104, date of approval: 20180104). The animals were maintained following the Guide for the Care and Use of Laboratory Animals. The surgical procedures performed in this study were approved by the Animal Ethics Committee, National Taiwan University Hospital, Taiwan.

A total of 27 rats were anesthetized with a 1:2 concentration of Zoletil (Virbac AH, Inc., Fort Worth, TX, USA) and Rompum (Bayer, Taipei, Taiwan) (1 mL/kg) via intraperitoneal injection. A 3 × 1 × 1 mm alveolar bone defect was created at the buccal site above the mental foramen. The alveolar bone defects were then filled with either the biomimetic hydroxyapatite microspheres (GHM-S group) or porous hydroxyapatite granules (“PURZER” SINBONE BONE REPLACEMENT, PURZER, Taipei, Taiwan: Sinbone group) and left without any filling material as a control; nine rats were used per material.

After 4, 8, and 12 weeks of implantation, the rats were sacrificed for the quantitative evaluation by using a high-resolution micro-computed tomography (μ-CT) (Skyscan1076, Kontich, Belgium). A semicircular region of interest (ROI) was accurately positioned above each defect for quantitative analysis (4 × 1 × 1 mm). The CTAnSkyscan software (https://www.bruker.com/products/microtomography/micro-ct-software/3dsuite.html) was used for the 3D analysis of alveolar bone regeneration and quantification of bone defect density from the reconstructed ROI images.

### 4.9. Histological Analysis

After μ-CT scanning, the specimens from the respective groups were fixed and decalcified. The specimens were then bisected and embedded in paraffin, sectioned at a thickness of 5 μm and placed on the glass slide. The slides were stained with hematoxylin and eosin (H&E) and Goldner’s Trichrome staining, then examined under a light microscope (IX71; Olympus, Tokyo, Japan) using Meta-Morph to visualize the formation of newly formed bone tissue.

### 4.10. Statistical Analysis

The data used in the figures are expressed as mean ± standard deviation (SD). Statistical analysis was done using a one-way ANOVA, the Bonferroni post hoc test is used for comparisons. Differences were considered significant at *p*-value less than 0.05. All analyses were performed using SPSS version 16.0 software (IBM, Taiwan, Taipei, Taiwan).

## 5. Conclusions

An adequate alveolar ridge dimensions to accommodate the implant is the prerequisite of a successful dental implant placement. In this study, gelatin/hydroxyapatite microsphere (GHM) was used to embed stromal cell-derived factor-1 (SDF-1), a well-characterized chemokine for attracting stem cells and a strong candidate for promoting regeneration. Our proposed biomimetic hydroxyapatite microspheres composite (GHM-S) has demonstrated an excellent biocompatibility with an efficient bone regeneration in vivo. This composite presents in this study may potentially be used as a bone substitute to regenerate alveolar bone defect and has therapeutic potential at other bone defects.

## Figures and Tables

**Table 1:** Primers used for osteogenic differentiation.

Gene Name	Forward Primer (5′–3′)	Reverse Primer (5′–3′)
ALPP	GAGAAGCCGGGACACAGTTC	CCTCCTCAACTGGGATGATGC
RUNX-2	TAGGCGCATTTCAGGTGCTT	GGTGTGGTAGTGAGTGGTGG
SP7	TAGGACTGTAGGACCGGAGC	CATAGTGAACTTCCTCCTGGGG
SPARC	ATTGACGGGTACCTCTCCCA	GAAAAAGCGGGTGGTGCAAT
BGLAP	CTCACACTCCTCGCCCTATTG	GCTTGGACACAAAGGCTGCAC
COL1a1	AGAGGTCGCCCTGGAGC	CAGGAACACCCTGTTCACCA
GAPDH	AATGGGCAGCCGTTAGGAAA	GCCCAATACGACCAAATCAGAG

ALPP: Alkaline phosphatase, RUNX-2: Runt-related transcription factor-2, SP7: Osterix, SPARC: Osteonectin, BGLAP: Osteocalcin, COL1a1: Type I collagen, GAPDH: Glyceraldehyde 3-phosphate dehydrogenase.
